# Differences in plasma microRNA content impair microRNA-based signature for breast cancer diagnosis in cohorts recruited from heterogeneous environmental sites

**DOI:** 10.1038/s41598-021-91278-0

**Published:** 2021-06-03

**Authors:** Jeanne P. Uyisenga, Ahmed Debit, Christophe Poulet, Pierre Frères, Aurélie Poncin, Jérôme Thiry, Leon Mutesa, Guy Jerusalem, Vincent Bours, Claire Josse

**Affiliations:** 1 Laboratory of Human Genetics, GIGA Research Institute, University of Liège, 11 avenue Hippocrate, B34 CHU Sart Tilman, 4000 Liège, Belgium; 2 Department of Biology, College of Science and Technology, University of Rwanda, Kigali, Rwanda; 3 Department of Medical Oncology, University Hospital of Liège, CHU Liège, Liège, Belgium; 4 Center for Human Genetics, College of Medicine and Health Sciences, University of Rwanda, Kigali, Rwanda; 5 Center of Human Genetics, University Hospital of Liège, CHU Liège, Liège, Belgium

**Keywords:** Cancer, Computational biology and bioinformatics, Genetics, Biomarkers, Medical research, Oncology

## Abstract

Circulating microRNAs are non-invasive biomarkers that can be used for breast cancer diagnosis. However, differences in cancer tissue microRNA expression are observed in populations with different genetic/environmental backgrounds. This work aims at checking if a previously identified diagnostic circulating microRNA signature is efficient in other genetic and environmental contexts, and if a universal circulating signature might be possible. Two populations are used: women recruited in Belgium and Rwanda. Breast cancer patients and healthy controls were recruited in both populations (Belgium: 143 primary breast cancers and 136 healthy controls; Rwanda: 82 primary breast cancers and 73 healthy controls; Ntot = 434), and cohorts with matched age and cancer subtypes were compared. Plasmatic microRNA profiling was performed by RT-qPCR. Random Forest was used to (1) evaluate the performances of the previously described breast cancer diagnostic tool identified in Belgian-recruited cohorts on Rwandan-recruited cohorts and vice versa; (2) define new diagnostic signatures common to both recruitment sites; (3) define new diagnostic signatures efficient in the Rwandan population. None of the circulating microRNA signatures identified is accurate enough to be used as a diagnostic test in both populations. However, accurate circulating microRNA signatures can be found for each specific population, when taken separately.

## Introduction

Breast cancer is the most commonly diagnosed and deadly malignancy in women in both developed and developing countries, with about 2.1 million cases and 627,000 deaths registered in 2018. It causes 25.1% of all cancer deaths each year in developed countries and is the leading cause of mortality among women in developing countries, with 14.3% of all deaths annually^1^.

In Belgium, about 11,000 new breast cancer cases in women and 2500 related deaths are registered every year (Belgian Cancer Registry^2^).

In Sub-Saharan Africa (SSA), breast cancer is becoming a major public health issue with a significant increase in its incidence, young age at diagnosis, and a high mortality rate as compared to developed parts of the world^3,4^. A recent study showed that more than 50% of all breast cancer women in Rwanda are younger than 50 years old, and more than 60% presented with advanced disease^5^.

The major challenge for optimizing the management of breast cancer is the early detection of the disease. Currently, mammography combined with invasive needle biopsy is used as a gold standard for breast cancer screening and allows early detection of the disease. However, it has some limitations; (a) exposure to X-ray radiation, (b) low sensitivity and specificity in young women or women with a high breast density^6^. Blood biomarkers such as carbohydrate antigen (CA 15.3) and carcino-embryogenic antigen (CEA) are useful for monitoring breast cancer treatment but lacks sensitivity for the detection of primary breast cancers^7^.

MicroRNAs are small non-coding RNAs of approximately 22 nucleotides long that regulate gene expression at the post-transcriptional level by binding to the 3′ untranslated region (UTR) of the target messenger RNAs. Studies have reported that microRNAs act as oncogenes or tumor suppressors and are aberrantly expressed in many cancers, including breast cancer^8–12^.

Circulating microRNAs have become promising non-invasive biomarkers of breast cancer. They can be easily measured in body fluids, including blood, plasma, and serum, where they remain highly stable^12^. Several microRNA profiling studies showed their potential as circulating biomarkers for diagnosis, prognosis, or prediction of treatment response in breast cancer pathology^13–16^.

Few studies have investigated the differential expression of microRNAs between different ethnic/ancestry groups. Some studies focusing on hypertension, diabetes, or uterine leiomyomas have shown differences of microRNAs between different populations. These differences were observed not only in the disease tissues but also in the healthy tissues^17–19^. In particular, a study comparing lymphoblastoid cell lines derived from 53 American subjects of European ancestry, and 54 people from Nigeria showed a high proportion of microRNAs that are differentially expressed in the two populations^20^. There are also few reports of microRNA expression differences in the breast cancer field. Nassar and colleagues compared breast cancer tissues of 45 Lebanese patients and 197 American patients, and highlighted ethnic differences^21^. Pollard et al*.* did the same kind of observation when comparing breast cancer tissues of 4 ethnic groups, i.e., British Caucasians, British Blacks, Nigerians, and Indians^22^. Two additional reports confirm such differences when comparing African American and European American patients^23,24^.

In our previous study of microRNA expression profiling of 188 microRNAs present in human plasma using reverse transcription quantitative polymerase chain reaction (RT-qPCR) and Random Forest-based analysis, we identified an 8 microRNA-based signature displaying high performance (AUC > 0.8) to detect breast cancers in a Belgian-recruited population^25^. The authors recommended its use in complement with mammography to identify early primary breast cancers. In the present study, we applied this “8 microRNA signature” on cohorts with a quite different genetic and environmental background as breast cancer patients and age-matched controls were recruited in Rwanda. Circulating microRNAs were quantified in their plasma. Diagnostic performances of several microRNA combinations, including the best performing that we called the “8 microRNA signature”, were determined using the same profiling and analysis method.

This work aimed at assessing if the previously identified circulating microRNA signature is efficient in another genetic and environmental context, and if a universal signature might be possible. The secondary objective was to find out an efficient signature to detect Rwandan breast cancer patients.

## Results

### Random forest

The random forest algorithm is a supervised machine learning classification method based on an ensemble of decision trees, that was first described by Breiman et al.^26^. In our study, Random Forest algorithms are used in a sequence of 3 main steps (see material and methods for details):

#### Feature selection

Its aim is to rank the plasmatic microRNAs according to the best discriminating power to correctly classify healthy women and primary breast cancers patients. It allows to reduce the number of microRNAs to those that contain more information than noise.

#### Building of random forest models

Its aim is to learn a prediction model from past observations. These observations are stocked in a “design” dataset that, in the present study, contains the plasmatic microRNA levels of breast cancer patients and healthy subjects that are known to be correctly classified based on the clinical diagnosis. A set of decision trees, called Random Forest models, is built from a bootstrap sample, which is a random selection of plasmatic microRNA levels drawn from the design dataset.

#### Validation of the models

Its aim is to predict the class—healthy or breast cancer- of new subjects, based on their plasmatic microRNA levels. During prediction, Random Forest outputs the class agreed by most of the individual trees. In this study, as the class of the subject in the validation dataset are also known, the classification performances of the models can be computed in terms of an area under the curve (AUC).

The first and second steps are performed on the well characterized “design” dataset. Of note, the dataset used to design and to validate (third step) the random forest models in this study are fully independent (see result section “Cohorts”).

Note that the first step is not mandatory and can be omitted when the number of features is small, or when the feature selection has been performed before. This is the case in the first result paragraph of this study (result section “Evaluation of the performances of the breast cancer diagnostic tool described by P. Frères and colleagues on Rwandan‑recruited cohorts”): the selection of the microRNAs forming the tested signatures was performed in our previous work^25^.

### Cohorts

The Belgian-recruited cohort contains 143 primary breast cancers and 136 healthy women^25^. The Rwandan-recruited cohort contains 82 primary breast cancers and 73 healthy women. In order to be used in the Random Forest procedure described above, this ensemble of subjects (N tot = 434) was split into four groups. Two groups were first created according to the recruitment site to allow the comparison of the two populations. Next, each site-specific group was split into two groups to allow the design of the microRNA signatures and their validation on independent cohorts, during the Random Forest procedure. The resulting four cohorts are called: *MATCHED-BE*; *MATCHED-RW*; *REST-BE* and *REST-RW*. The suffix -*BE* or -*RW* will be used according to the recruitment site. The *MATCHED-BE* and *MATCHED-RW* cohorts are matched in terms of age, breast cancer status, breast cancer subtype and subject number to allow a controlled comparison of the two populations. Each of them contains 55 healthy women, and 55 primary invasive breast cancer patients. The detailed information of those *MATCHED* cohorts is available in Table 1. The *REST-BE* cohort contains the remaining patients of the cohort described in Frères et al.: 88 primary breast cancer patients and 81 healthy women^25^. The *REST-RW* cohort contains the remaining 27 primary breast cancer patients and 18 healthy women recruited in Rwanda. The detailed information of the *REST* cohorts is available in Table S2.

**Table 1:** Matched cohorts – Clinical and pathological data and tumor characteristics of study participants.

	Belgian-recruitment site (n = 110)		Rwandan-recruitment site (n = 110)	
	Primary breast cancers (n = 55)	Healthy women (n = 55)	Primary breast cancers (n = 55)	Healthy women (n = 55)
Median age (range) (y)	45 (26–68)	43 (27–63)	45 (27–65)	46 (31–64)
Estrogen receptor [n (%)]	34 (61.8)		34 (61.8)	
HER2 [n (%)]	15 (27.3)		15 (27.3)	
**Molecular subtype [n (%)]**				
NA	0		0	
1 = ER + /HER2-	26 (47.3)		26 (47.3)	
2 = ER + /HER2 +	8 (14.5)		8 (14.5)	
3 = ER-/HER2 +	7 (12.7)		7 (12.7)	
4 = TN	14 (25.5)		14 (25.5)	
**Lymph node involvement [n (%)]**				
NA	0 (0)		22 (40)	
1	29 (52.7)		30 (54.5)	
0	26 (47.3)		3 (5.5)	
**Stage [n (%)]**				
NA	0 (0)		6 (10.9)	
1	9 (16.4)		1 (1.8)	
2	33 (60)		13 (23.6)	
3	13 (23.6)		35 (63.6)	
**Histologic subtype [n (%)]**				
NA	0 (0)		1 (1.8)	
IDC	46 (83.6)		49 (89)	
ILC	8 (14.5)		2 (3.6)	
other	1 (1.8)		3 (5.5)	

### Study summary

**Figure 1:**
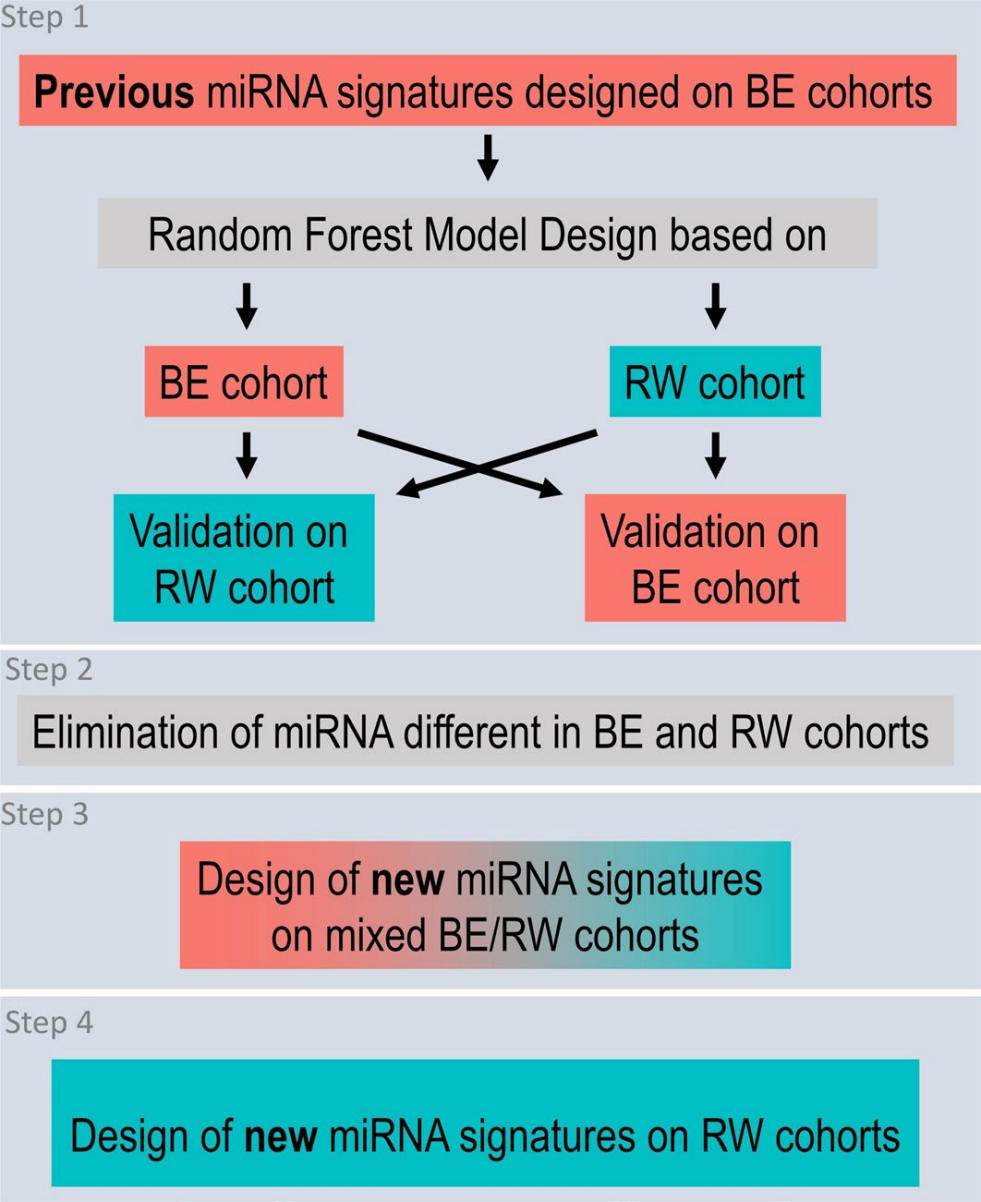
Overview of the major steps of the study.

The overview of the major steps of the study is presented in Fig. 1. We first considered the results from our previously work^25^ and evaluated the diagnostic accuracy of 13 circulating microRNA signatures designed before in two different population-cohorts of breast cancer patients and age-matched healthy controls with different genetic and environmental backgrounds.

**Figure 2:**
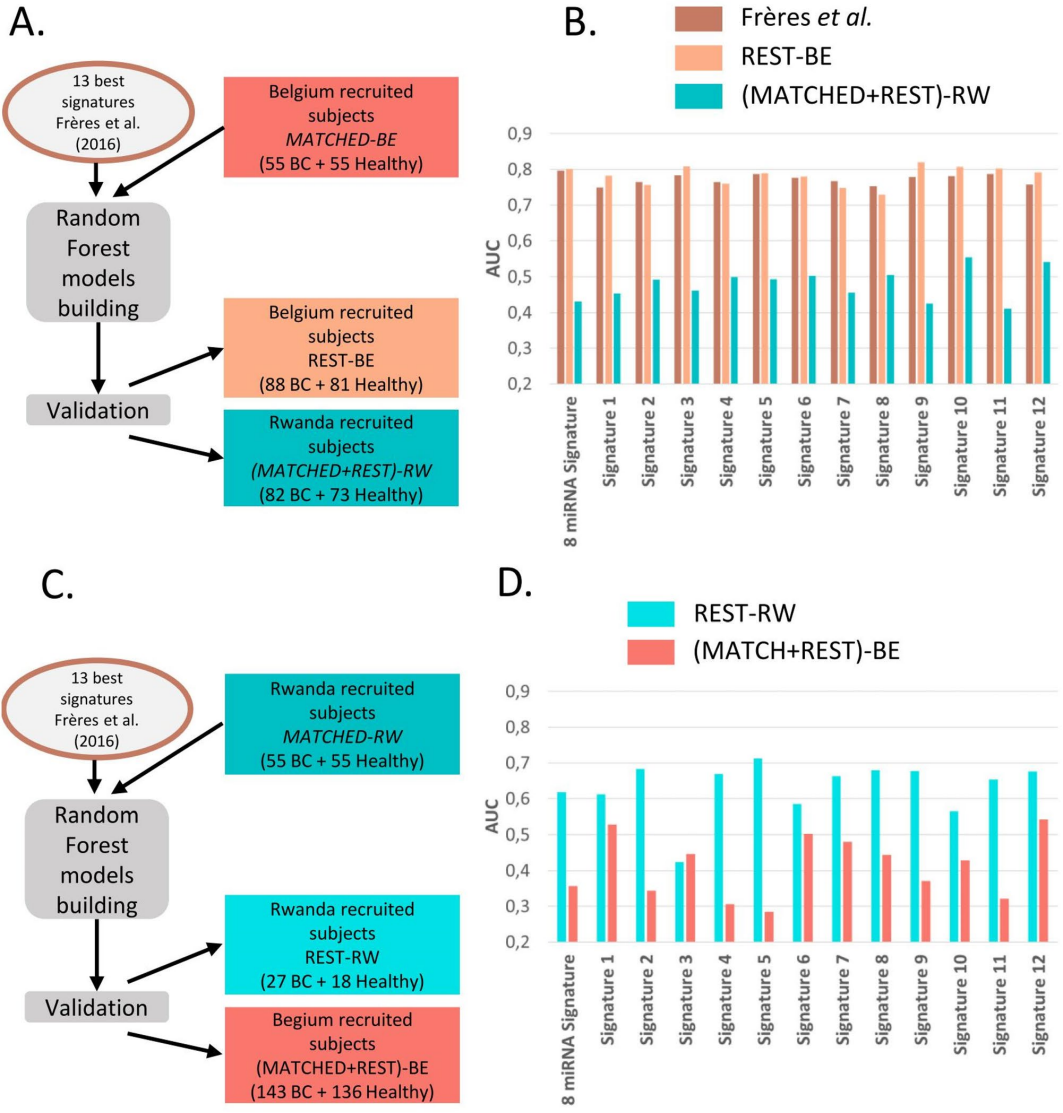
**The diagnostic microRNA signatures designed on cohort from one recruitment site are not well performing on cohorts from a different recruitment site**. (**A**) Flowchart of the Random Forest procedure: Belgian-recruited subjects are used to design the models, and validation is performed either on Belgian or Rwandan-recruited subjects. (**B**) Performances of the 13 signatures on two validation datasets containing subjects from Rwanda [(MATCHED + REST)-RW] or Belgium [REST-BE]. (**C**) Flowchart of the Random Forest procedure: Rwandan-recruited subjects are used to design the models, and validation is performed either on Belgian or Rwandan-recruited subjects. (**D**) Performances of the 13 signatures on two validation datasets containing subjects from Rwanda [REST-RW] or Belgium [(MATCHED + REST)-BE].

**Figure 3:**
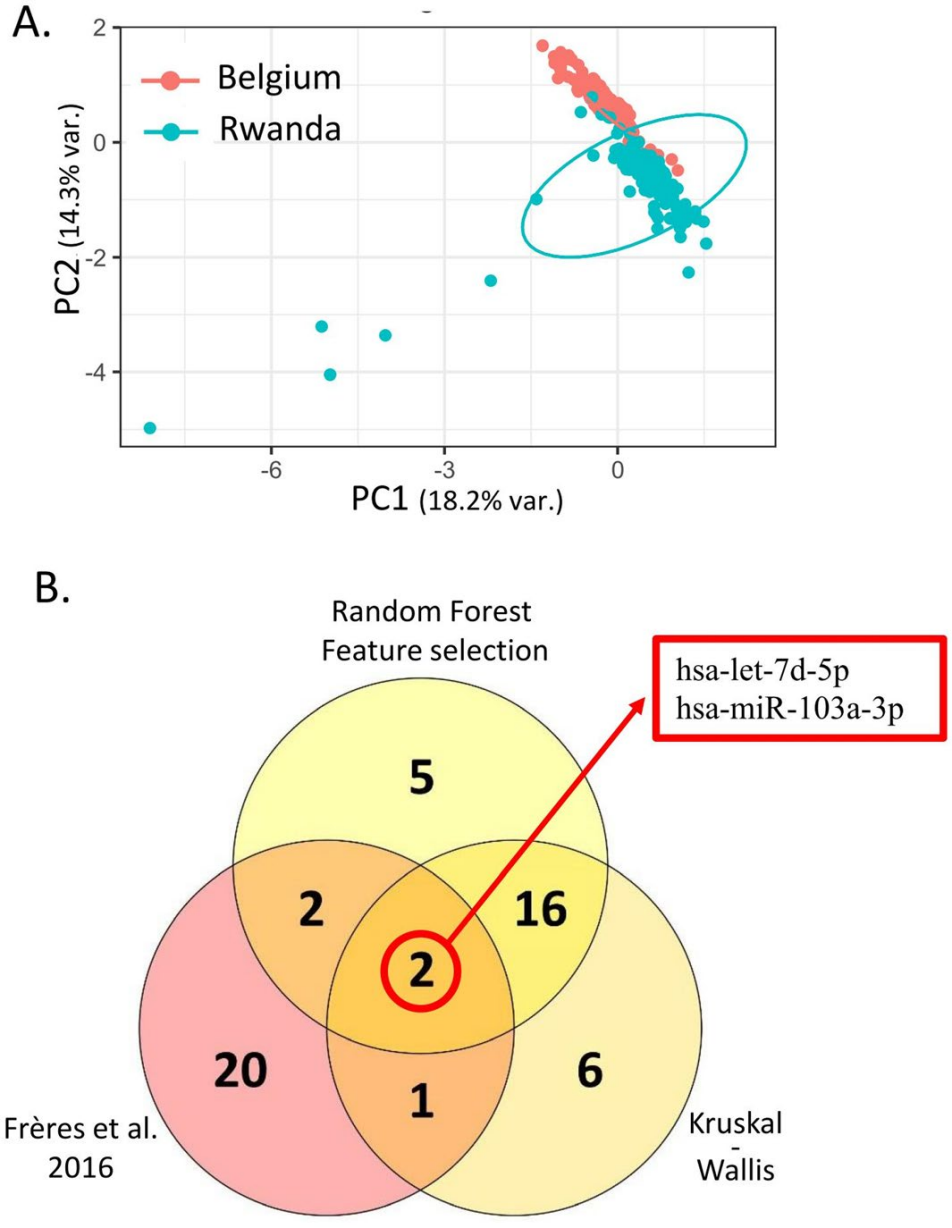
**Circulating microRNA contents are different in Belgian- and Rwandan-recruited populations.** (**A**) Principal Component Analysis performed on the plasmatic microRNAs from cohorts MATCHED-BE (red) and MATCHED-RW (blue) shows that the two populations display distinct profiles. (**B**) The best 25 circulating microRNAs able to discriminate Belgium-from Rwanda-recruited women were determined either by random forest feature selection or by Kruskal–Wallis statistical test and are represented in the two yellow sets. The 25 circulating microRNAs that were best performing to discriminate healthy women from breast cancer patients in a Belgian-recruited population as determined in the publication Frères et al*.* are represented in the pink set. The Venn diagram is showing the intersection of these three groups, highlighting hsa-let-7d-5p and hsa-miR-103a-3p that are able to both discriminate healthy/cancer and Rwanda/Belgium women.

**Figure 4:**
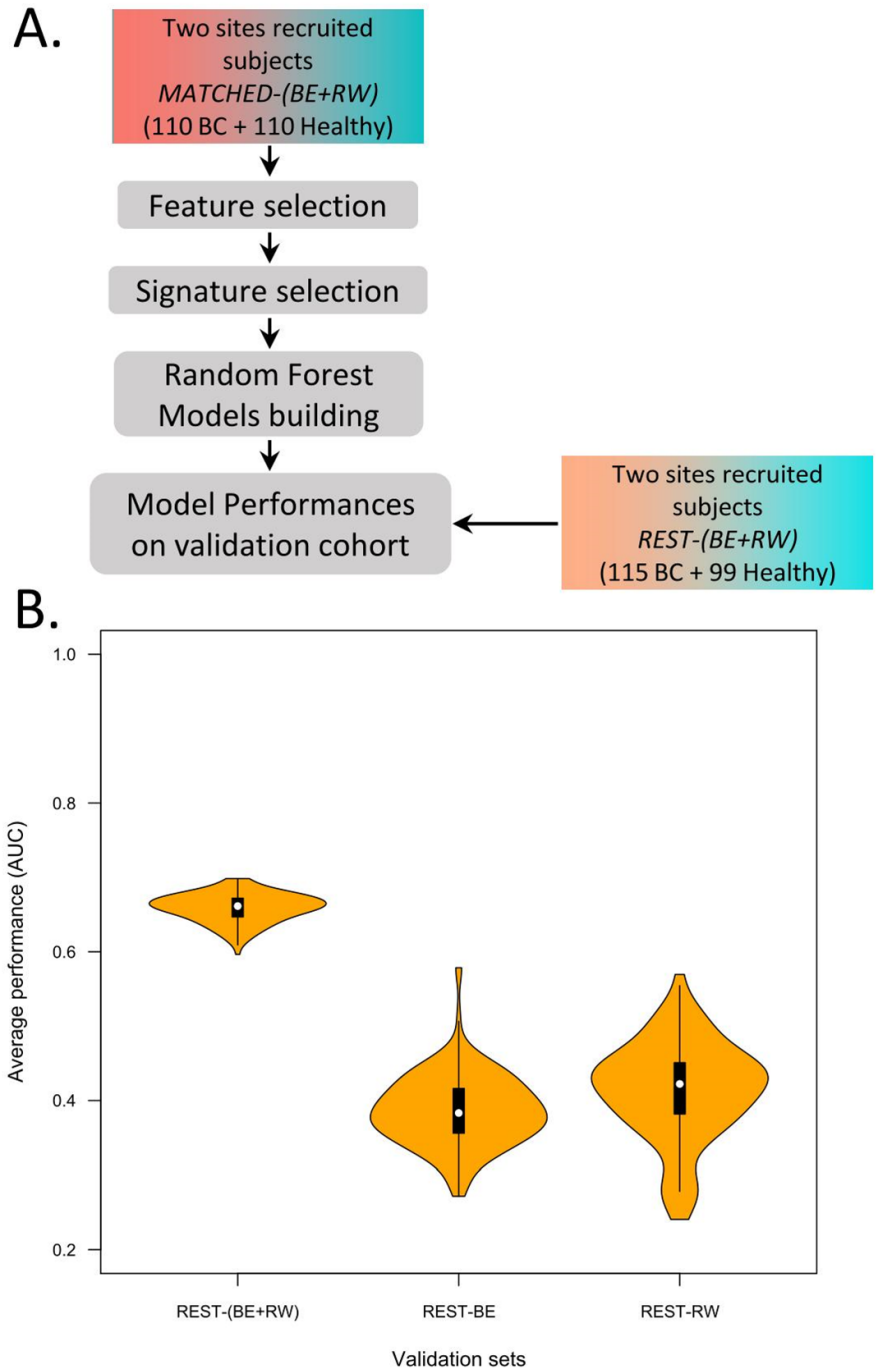
**Diagnostic circulating microRNA signature designed in a mixed recruitment site cohort are inefficient.** (**A**) Flowchart of the Random Forest procedure: both design and validation cohorts contain patients from the two recruitment sites. (**B**) Performances of the 163 signatures on independent validation dataset containing subjects from Rwanda and Belgium recruitment sites. REST-BE and REST-RW are the two sub-cohorts of breast cancers of REST-(BE + RW). REST-(BE + RW) has been normalized as a whole.

**Figure 5:**
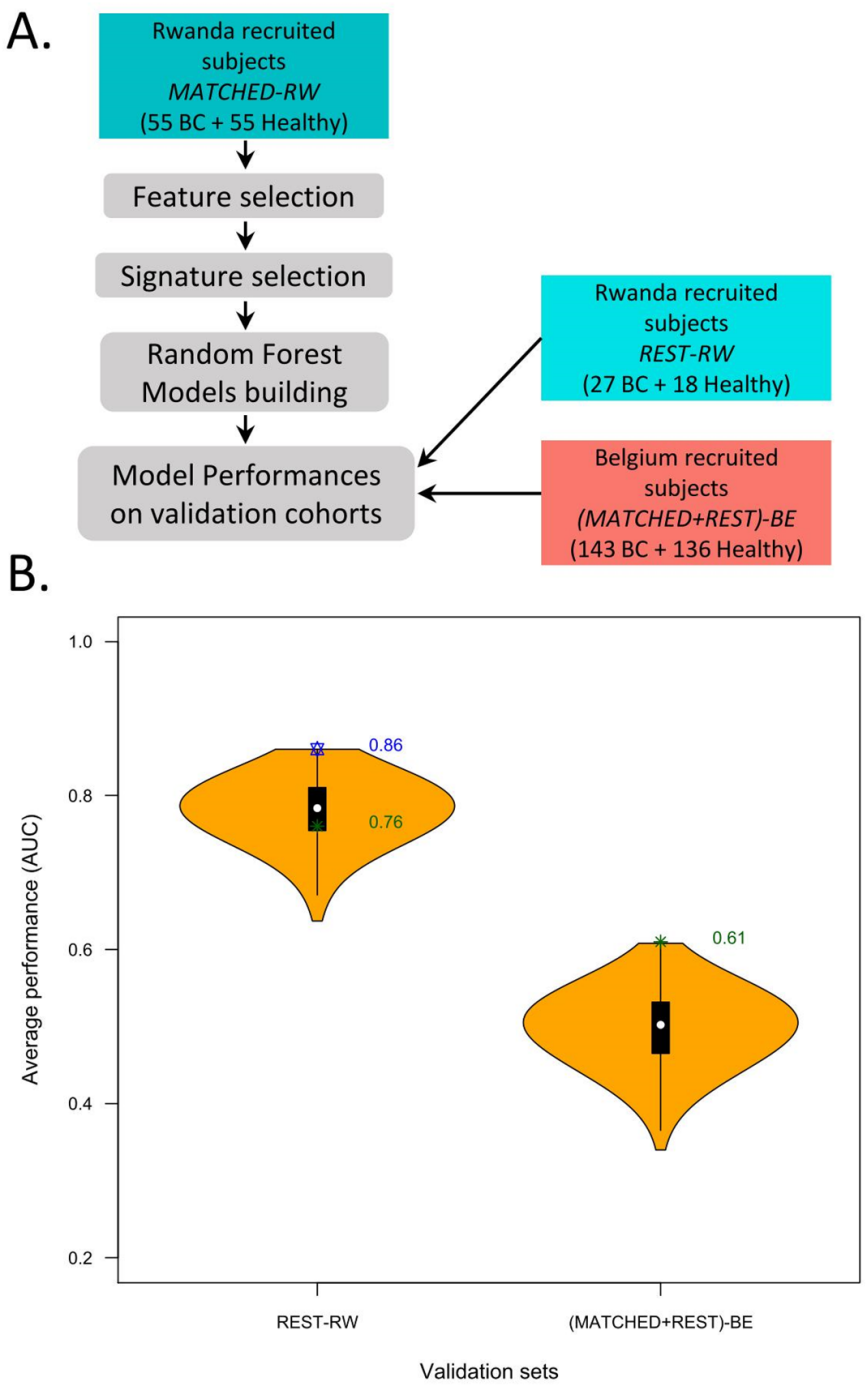
**Diagnostic circulating microRNA signatures are efficient only when there are selected in a single site recruitment population.** (**A**) Flowchart of the Random Forest procedure. (**B**) Performances of the signatures designed on a Rwandan dataset on two independent validation datasets containing either subjects from Rwanda (REST-RW) or from Belgium (MATCHED + REST)-BE. The coloured AUC values correspond to the following: blue: the most performing signature on the Rwandan-recruited validation cohort; and green: the most performing signature when validated on a Belgian-recruited cohort.

**Figure 6:**
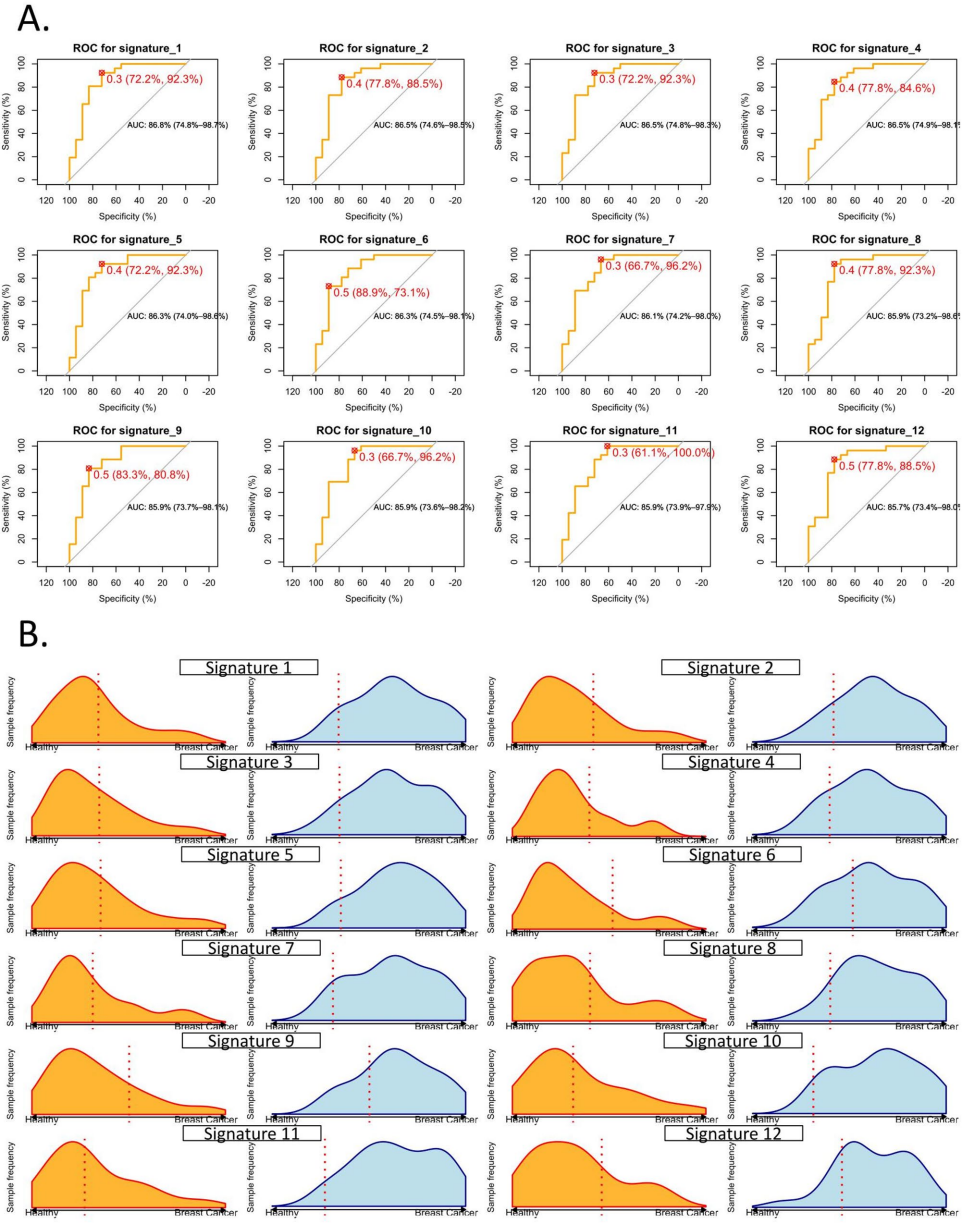
**Performances of the 12 best circulating microRNA signatures able to discriminate breast cancers from healthy women in Rwandan-recruited subjects.** (**A**) ROC curve analysis, optimal cut-off, and corresponding specificity and sensibility (red brackets) of the top signatures. Optimal cut-offs are calculated using the Youden index and are displayed in red. The confidence intervals of the AUC values are displayed in black brackets (**B**) Model outcome distributions for the breast cancer and healthy subjects. The x-axis corresponds to the model predictions. The dashed line represents the chosen threshold used to compute the sensitivity and specificity values for each group. Orange distribution contains healthy subjects; breast cancer subject distributions are displayed in blue. Names of the signatures are referring to Table 3.

To do this, we measured the levels of 175 circulating microRNAs in a cohort of women recruited in Rwanda with the same RT-qPCR profiling method. These data were analyzed using the same Random Forest-based method.

i. Step 1—result section "Evaluation of the performances of the breast cancer diagnostic tool described by P. Frères and colleagues on Rwandan-recruited cohorts ": For the 13 previously defined signatures, new random forest models were designed and validated using the following combinations: (i) design of the random forest models on a cohort recruited in Belgium and validation on a separate Belgian cohort and on a second cohort recruited in Rwanda (Fig. 2A, B) ; (ii) design of the random forest models on the Rwandan-recruited cohort and validation on a separate Rwandan cohort, and on a second Belgian-recruited cohort (Fig. 2C, D).
ii. Step 2—result section  "The profile of circulating microRNA levels is different in the Belgian and Rwandan recruitment sites": Circulating microRNAs significantly different in Belgian and Rwandan women are searched to be removed from the analysis performed in Step 3 (Fig. 3).
iii. Step 3—result section  "Defining new signatures able to discriminate patients from healthy women in both recruitment sites": Design of new diagnostic circulating microRNA signatures and the corresponding random forest models on a mixed cohort of Rwanda- and Belgian-recruited patients, and validation on a separate mixed cohort (Fig. 4).
iv. Step 4—result section  "Defining new signatures able to discriminate patients from healthy women in Rwanda only": Design of new circulating microRNA signatures and the corresponding random forest models, considering the Rwandan-recruited cohorts only, for both design and validation of the signatures (Figs. 5 and 6).

For both groups, healthy controls with matched age were also considered. Patients with matched age and breast cancer subtypes were only included in the analysis.

### Evaluation of the performances of the breast cancer diagnostic tool described by P. Frères and colleagues on Rwandan-recruited cohorts^25^

The flowchart of this experiment is depicted in Fig. 2A. Random Forest models of the “8 microRNA signature” were first designed on the microRNA dataset corresponding to the Belgian recruited cohort *MATCHED-BE*. Next, the Random Forest models were applied on two validation datasets containing microRNA levels of subjects from the two recruitment sites: *REST-BE* and the fusion of the *MATCHED-RW* and *REST-RW* (Table S3). The resulting AUC of the “8 microRNA signature” are represented in Fig. 2B, first boxes. The AUC obtained in our previous study is also plotted as reference. The AUC of the “8 microRNA signature” designed on the Belgian-recruited population (*REST-BE*) is quite similar to the value described in Frères et al. (2016), which is expected. The slight difference observed is explained by the fact that the models were designed in a subgroup of the cohorts used in our previous work. By contrast, the AUC obtained in the Rwandan-recruited population is drastically reduced compared to the performances obtained in the Belgian-recruited population.

Besides the best performing “8 microRNA signature”, twelve other alternative signatures with comparable performances were also found in our previous work (unpublished results). This can be explained by the fact that many microRNAs might be deregulated in the same manner in a particular disease condition, leading to many correlated circulating microRNA level variations. In consequence, one microRNA may be replaced by another microRNA in a specific signature^25^. The performances of 12 other signatures displaying a mean AUC > 0.70 in the Belgian population (Table 2), were also tested on the different cohorts of this study (Fig. 2B). The 12 alternative signatures display the same variation profile of performances in the different cohorts than the “8 microRNA signature”: none of them displayed an AUC > 0.6 in the Rwandan-recruited cohorts when the models were computed in subjects of the Belgian recruitment site. The resulting AUC of these signatures in each dataset is available in Table S4.

**Table 2:** Circulating microRNA composition of the tested signatures.

Signature Name	MicroRNA signature composition
The “8 microRNA Signature”	hsa-miR-16-5p + hsa-let-7d-5p + hsa-miR-103a-3p + hsa-miR-107 + hsa-miR-148a-3p + hsa-let-7i-5p + hsa-miR-19b-3p + hsa-miR-22-5p
Signature_1	hsa-let-7d-5p + hsa-miR-16-5p + hsa-miR-103a-3p + hsa-miR-199a-5p
Signature_2	hsa-let-7d-5p + hsa-miR-16-5p + hsa-miR-103a-3p + hsa-miR-22-3p + hsa-miR-30b-5p
Signature_3	hsa-let-7d-5p + hsa-miR-32-5p + hsa-miR-199a-5p + hsa-miR-142-3p + hsa-miR-22-5p
Signature_4	hsa-let-7d-5p + hsa-miR-148a-3p + hsa-let-7i-5p + hsa-miR-199a-5p + hsa-miR-451a
Signature_5	hsa-let-7d-5p + hsa-miR-148a-3p + hsa-let-7f.-1-3p + hsa-miR-199a-5p + hsa-miR-32-5p
Signature_6	hsa-miR-16-5p + hsa-let-7d-5p + hsa-miR-103a-3p + hsa-miR-148a-3p + hsa-let-7f.-1-3p + hsa-miR-32-5p
Signature_7	hsa-miR-16-5p + hsa-let-7d-5p + hsa-let-7i-5p + hsa-miR-19a-3p + hsa-let-7f.-1-3p + hsa-miR-1-3p
Signature_8	hsa-miR-16-5p + hsa-let-7i-5p + hsa-miR-19a-3p + hsa-miR-451a + hsa-miR-19b-3p + hsa-miR-32-5p
Signature_9	hsa-miR-16-5p + hsa-let-7d-5p + hsa-miR-103a-3p + hsa-miR-148a-3p + hsa-miR-19a-3p + hsa-miR-199a-5p + hsa-miR-22-3p
Signature_10	hsa-miR-16-5p + hsa-let-7d-5p + hsa-miR-103a-3p + hsa-miR-20a-5p + hsa-let-7i-5p + hsa-miR-1-3p + hsa-miR-32-5p
Signature_11	hsa-miR-16-5p + hsa-let-7d-5p + hsa-miR-103a-3p + hsa-miR-148a-3p + hsa-miR-93-5p + hsa-miR-451a + hsa-miR-1-3p + hsa-miR-22-5p
Signature_12	hsa-miR-16-5p + hsa-let-7d-5p + hsa-miR-103a-3p + hsa-miR-20a-5p + hsa-let-7f.-1-3p + hsa-miR-30b-5p + hsa-miR-590-5p + hsa-miR-22-3p

Alternatively, the MATCHED-RW cohort was used to model the Random Forest trees corresponding to the 13 signatures (Table 2), and those models were validated on dataset containing subjects of different origin: REST-RW, and the fusion of MATCHED-BE and REST-BE (Table S3 and Fig. 2C). The 13 signatures display lower performances in Rwandan-recruited population than what was observed when the models were designed in Belgian-recruited population (compare REST-BE in Fig. 2B and REST-RW in Fig. 2D). And the performances were again lower when the cohort used for the random forest models validation is recruited in the other site than the cohort used for the design of the models (Fig. 2D). Details of the signature performances in each cohort can be found in Table S4.

Quite similar results were obtained when we designed and validated complete new microRNAs signatures, instead of new random forest models only, using the same scheme for the choice of the design and the validation cohorts (data not shown).

### The profile of circulating microRNA levels is different in the Belgian and Rwandan recruitment sites

These results suggest that the circulating microRNA levels are different in the Belgian and Rwandan recruitment sites. Indeed, the Fig. 3A shows that the two sites of recruitments can be distinguished by the two first components of a principal component analysis.

If some microRNAs have the simultaneous potential to allow discrimination of breast cancer patients from healthy women, but also to allow discrimination of Belgian-recruited population from Rwandan-recruited population, they need to be removed from the dataset in order to be able to find signatures working on both recruitment sites.

We, thus, performed a Random Forest feature selection process to find out the 25 best performing circulating microRNAs able to discriminate Belgian-recruited women from Rwandan-recruited women (N = 434). In parallel we did the same using a conventional statistical test, Kruskal–Wallis. Finally, we compared the intersection of those microRNAs with the 25 microRNAs determined in our previous work to be the best discriminating for cancer/healthy plasma in Belgian-recruited women (Fig. 3B). By this method, we determined that the microRNAs hsa-let-7d-5p and hsa-miR-103a-3p need to be removed from the dataset to proceed to our next analysis step. They were also the only two microRNAs appearing as discriminating the Belgian from the Rwandan women when only healthy women were compared, or when only breast cancer patients were compared (data not shown).

### Defining new signatures able to discriminate patients from healthy women in both recruitment sites

The three steps of the Random Forest procedure were then applied on the new dataset excluding hsa-let-7d-5p and hsa-miR-103a-3p, as depicted in Fig. 4A. The design dataset for Random Forest procedure contains the patients from both recruitment sites: it is the fusion of the *MATCHED-BE* and *MATCHED-RW* cohorts. The validation dataset also contains subjects from the two recruitment sites (fusion of the *REST-BE* and *REST-RW*—see Table S3).

The feature selection result—depicted in the Figure S1—allowed the identification of 13 microRNAs that are the most important and robust in distinguishing the cancer from the healthy subjects. The performances of all the possible combinations of these 13 microRNAs generated (2^13–1 = 8191) were calculated on the design cohort, and 163 combinations with AUC ≥ 0.82 (Table S5) were identified. The performances of those 163 combinations were next evaluated on the validation datasets (Fig. 4B). The AUC values in the total validation cohort are ranging from 0.596 to 0.698 (Standard deviation, SD = 0.14). However, these values are dropping in the *REST-BE* (0.272–0.578; SD = 0.025) and *REST-RW* (0.240–0.569; SD = 0.044) sub-cohorts, when they are considered separately.

In conclusion, none of the circulating microRNA signature designed on dataset containing heterogeneous recruitment site patients is efficient.

### Defining new signatures able to discriminate patients from healthy women in Rwanda only

As circulating microRNA signatures established on patients recruited on mixed genetic and environmental background were not efficient enough to be used as diagnostic tool, Rwandan-only subjects were used in this part of the work to design new signatures able to diagnose breast cancers in this population.

A design dataset containing the circulating microRNA data of Rwandan-only recruited subjects—*MATCHED-RW*—was used in this Random Forest procedure, which is depicted in Fig. 5A. Next the validation of the models was performed on two datasets that contain either the microRNA data of Rwandan-recruited subjects (*REST-RW*) or Belgian-recruited subjects (REST-BE) (Table S3).

The feature selection allowed the identification of 17 microRNAs efficient to discriminate healthy women from cancer patients. The performances in terms of AUC of the 131.071 combinations of those 17 microRNAs (2^17–1 = 131,071) were first evaluated on a subset of the same design cohort (test cohort, see material and methods). The 995 signatures that displayed AUC ≥ 0.94 were selected to be next validated on two datasets constructed with microRNA data from patients recruited on the two different recruitment sites. Figure 5B is depicting the results obtained.

Among the 17 identified microRNAs from the feature selection in the Rwandan design dataset, the expression of hsa-miR-342-3p and hsa-miR-185-5p were not measured in the Belgian validation dataset. Consequently, all the signatures among the 995 containing at least one of those microRNAs were discarded when evaluated on the Belgian cohort. Only 623 signatures were validated on the Belgian cohort.

The best signature on the Rwandan validation cohort displays an AUC = 0.86 and is highlighted in blue in Fig. 5. However, as it contains the hsa-miR-342-3p, this signature was not validated in the Belgian dataset.

Among the 623 signatures validated on the Belgian cohort, the best performing display poor performance (AUC = 0.61), showing again that only cohorts from a single recruitment site can be used to design powerful circulating diagnostic microRNA signatures.

Eleven alternative signatures displaying good performances on the Rwandan validation cohort were also selected for the next steps analyses. In order to calculate their sensitivities and specificities, the optimal cut-off of the ROC curves were calculated according to the Youden index^27^. Table 3 reports the AUC of 12 best signatures on the Rwandan-recruited cohort, and their corresponding specificity and sensitivity metrics. More details about their composition in circulating microRNAs are available in Table S6. The corresponding receiver operating characteristics (ROC) curves are displayed in Fig. 6A. The 12 signatures were then used to compute the probability of each subject of the Rwandan-recruited validation cohort to be classified into healthy or cancer groups, and the distribution of those probabilities is depicted in Fig. 6B.

**Table 3:** The 12 top most performing signatures identified in the independent Rwandan-recruited validation cohort REST-RW.

	AUC	Sensitivity	Specificity	Number of microRNAs per signature
RW_1	0.8675	0.92	0.72	13
RW_2	0.8654	0.88	0.78	13
RW_3	0.8654	0.92	0.72	11
RW_4	0.8654	0.85	0.78	13
RW_5	0.8632	0.92	0.72	13
RW_6	0.8632	0.73	0.89	12
RW_7	0.8611	0.96	0.67	11
RW_8	0.8590	0.92	0.78	8
RW_9	0.8590	0.81	0.83	11
RW_10	0.8590	0.96	0.67	10
RW_11	0.8590	1	0.61	10
RW_12	0.8568	0.88	0.78	8

Among the 12 best performing diagnostic signatures for Rwandan-recruited cohort, the 4 microRNAs hsa-let-7a-5p, hsa-miR-126-3p, hsa-miR-150-5p, and hsa-miR-940 appear to be particularly important to distinguish healthy women from breast cancer patients, as they appear in all the 12 signatures.

## Discussion

An important improvement in the management of breast cancer would result from the discovery of sensitive and specific minimally invasive biomarkers to detect breast cancers at early stage^28^. A good biomarker should be easily accessible and non-invasive, sensitive enough to detect early tumors in almost all patients and absent in healthy women. None of the current diagnostic biomarkers used for breast cancer detection meets the above-cited criteria^29^. However, circulating microRNAs have been identified in serum and plasma, and are increasingly recognized as powerful disease biomarkers for breast cancers^30,31^.

In our previous work, we identified an 8 microRNA-based signature, quantified by RT-qPCR in plasma. This signature can discriminate breast cancer patients from healthy controls in Belgian-recruited women with high performance. We have also identified other alternative microRNA signatures with an AUC > 0.8. Among them, an 11 microRNA signature was validated in an independent cohort of 54 serum samples from Singaporeans of Chinese ancestry and gave an AUC of 0.77^25^. However, it is still largely unknown if the signatures that are established in a specific population can be applied to other groups from different ancestries.

In this study, we found that the diagnostic circulating microRNA signatures perform better on their own geographical and environmental recruitment site, and that their performances decrease when they are established on the recruitment group from a different continent. We also found that the plasmatic microRNA content of the Belgian and Rwandan population is highly different. In particular, 2 microRNAs that are part of most of the best performing microRNA signatures described by Frères et al., namely let-7d-5p and miR-103a-3p, are also able to discriminate the two ethnic groups. This might explain the observed performance differences of the diagnostic signatures between cohorts of different origins.

However, even when these two microRNAs are excluded from the analysis, none of the 163 newly designed signatures on cohort from mixed origins could differentiate breast cancers from healthy controls in either Rwandan or Belgian cohorts with sufficient performance for a clinical test. In opposite, highly performant circulating diagnostic signatures can be found in a homogeneous population, as illustrated by the determination of 12 newly designed signatures in the Rwandan cohorts, and by the results of Frères et al. in the Belgian cohort.

Indeed, when we considered data from the Rwanda-only recruited subjects, the feature selection process highlighted 17 efficient microRNAs to discriminate healthy women from breast cancers patients. Combining them, we were able to identify 12 microRNA signatures that could be used to diagnose breast cancer in Rwanda, all displaying AUC > 0.86. Among the 17 plasmatic microRNAs, four of them seem to be particularly important as they are part of all the 12 best signatures. These four microRNAs, let-7a-5p, miR-126-3p, miR-150-5p and miR-940, are known to have deregulated expression in breast cancer tissues, and are involved in molecular pathways leading to breast cancer development or progression^32–36^.

The performances of the screening mammography are described in many publications with sensitivity values ranging from 87 to 70%, and specificity values ranging from 97 to 89%^37–39^. The 12 circulating microRNA diagnostic signatures selected to detect breast cancer in Rwandan women display higher sensibility than mammography, but lower specificities, meaning that more false positive cases might be found using this technique. However, the signature 8 seems to be the best compromise with its specificity of 78% and sensitivity of 92%—which are near of the range of the mammography values—and composed of only 8 circulating microRNAs. As this number is low, their quantification by RT-qPCR, a technique often used in clinical routine, would be easy to implement in clinical use. Indeed, we aimed at selecting short combinations of biomarkers to facilitate their transfer towards the medical practice.

To date, few comprehensive investigations of population differences in microRNA content, either at the tissue or at the circulating levels, were conducted in breast cancer.

At the circulating level, Zhao et al. investigated plasmatic microRNA levels in breast cancer patients and matched controls from different ethnic origins, and found that 31 microRNAs were differentially expressed in plasma of 10 Caucasian Americans and 10 African Americans with two microRNAs overlapping between the two ethnic groups. However, the patients and controls in this-cited study were recruited from the same living environment^40^.

At the breast cancer tissue level, Pollard et al. analyzed the microRNA expression of four different ethnic groups (British Blacks, British Caucasians, Indians and Nigerians) and confirmed that microRNAs are differentially expressed in their tumor tissues. The authors found that miR-140-5p, miR-194 and miR-423-5p were upregulated in Nigerians compared with the other ethnic groups. They concluded that these disparities in microRNA expression between African and European descent women could be linked to the differences in the frequency of the tumor molecular subtypes in those ethnic groups. Indeed, breast tumors in sub-Saharan African women are predominantly hormone negative and triple negative subtypes while breast tumors in European women are predominantly hormone positive and luminal subtypes, and distinct molecular pathways are involved in those breast cancer subtypes^22^. This drawback in the cohort selection was avoided in our study, as the enrolled subjects of the two populations were matched in terms of breast cancer molecular subtypes.

Another study showed that European, African, and Asian subjects display population-specific genetic variations in microRNA genes associated with cancer risk. Those genetic variations may also affect microRNA expression and contribute to observed population differences in health disparities in multiple forms of cancers, including breast cancers^41^.

Our results are in line with these few studies showing differences in microRNA content in tissues and/or circulating compartments between specific ethnic groups and suggest that the differences in the plasmatic microRNA content observed between Belgian and Rwandan women could be linked to their genetic background, living environment and/or life style. However, the size of the two populations our study is too small to allow conclusions across the human populations, focused in European and African ancestries.

Among limitations of this study, we can cite that the dataset used for the design of the signatures were over-represented by the ER + HER− (47.3%) compared to the other subtypes (ER + HER + : 14.5%, ER-HER + : 12.7%, and ER-HER−: 25.5%). In such setting, the signatures can be biased to the most representative subtype. Breast cancer patients recruited in Rwanda had also more advanced tumor stages and more frequent lymph nodes metastasis compared to their Belgian counterparts. Moreover, the metastasis detection was not systematically performed in Rwanda. Yet, a number of studies have shown that microRNAs are differentially expressed between early, advanced and metastatic stages in breast tumor tissues^42–45^. We have also observed differences in the plasmatic microRNA content between metastatic and primary breast cancers, in Belgian-recruited patients (unpublished results). Besides, there is a lack of data on tumor subtypes in the Rwanda validation cohort (Table S2). Our study would have been improved if we had them, as we could test the performances of the 12 Rwanda-specific signatures on sub-cohorts of different tumor subtypes. In addition, we do not have precise information about the bloodline of the recruited subjects, but only their living country. We do not have any information either about inflammatory, autoimmune or chronic diseases of the recruited patients/controls, that might also affect the results obtained. Nevertheless, this is the first study providing valuable information on plasmatic microRNA content in large cohorts of breast cancer patients from different ethnicities with quite different genetic background and living environment.

In conclusion, efficient plasmatic microRNA signatures can be found to diagnose breast cancers, but their performances only reach the level needed for clinical use when the signature are designed on homogeneous geographical and environmental groups. This is probably due to high disparities in plasmatic microRNA content between populations, that are consequences of genetic and/or environmental differences. Further studies would be needed to distinguish those two aspects, and to compare other ethnic groups. Even so, the results of this study are suggesting that finding a universal circulating microRNA signature is highly unlikely.

Moreover, we describe several efficient plasmatic microRNA signatures to diagnose breast cancers in Rwandan women. The more suitable for clinical use display, when tested on an independent cohort, AUC of 86% (sensitivity = 92% and specificity = 78%). This short biomarker signature contains only 8 microRNAs, which can be measured in the plasma—in a non-invasive procedure – by RT-qPCR—which is a simple technique routinely used in hospitals. These features could favor an easy transfer to the medical practice.

## Materials and methods

### Patients and healthy controls

Rwandan recruitment site: patients and control healthy women with matched-age were recruited at three teaching hospitals in Rwanda: Butare Teaching Hospital (CHUB), Kigali Teaching Hospital (CHUK) and Rwanda Military Hospital (RMH) from May 2016 to May 2018.

Belgian recruitment site: the recruitment was described in^25^. Briefly, patients and healthy women were recruited prospectively at Centre Hospitalier Universitaire CHU of Liège and Clinic Saint-Vincent (Liège, Belgium) from July 2011 to September 2014.

In both cohorts, healthy women displayed normal mammograms and breast cancers subtypes were defined using routine immunohistochemistry for estrogen receptor (ER), progesterone receptor (PgR), and human growth factor receptor 2 (HER2). In the Belgian cohort only, HER2 status was confirmed by fluorescent in situ hybridization (FISH).

### Ethical consideration

The study was conducted in accordance of the Declaration of Helsinki for both recruitment sites. In Rwanda, the study was conducted with the approval of the Institutional Review Board (IRB) of the College of Medicine and Health Sciences (CMHS) at University of Rwanda (No: 156/CMHS IRB/2016) and the ethical committee of each hospital: CHUK Clinical Research Ethical Committee (Ref.: EC/CHUK/089/2016); CHUB Research Ethical Committee (Ref.: CHUB/DG/SA/5/869/2016) and RMH Research Ethical Committee (Ref.: EC/RMH/051/2016). from the Institutional. In Belgium, this study was approved by the Ethics Committee of the University Hospital of Liège (Ref.: B70720109893/2018/191).

All study participants signed the written informed consent prior to enrollment, in both recruitment sites.

### Plasma samples

Blood samples from Rwanda recruitment site was performed using the same protocol than used previously for the Belgian cohort^25^. Blood were collected in EDTA tubes and double centrifuged within 1h of collection to get plasma. The two centrifugations were done at 4 °C during 10 min, at 815 × g and 2500 × g respectively. Plasma were stored at −80 °C until use.

### Total RNA extraction from plasma

RNA extractions from blood samples of the Rwanda recruitment site were performed using the same protocol than used previously for the Belgian cohort^25^. The degree of hemolysis of all plasma samples was evaluated prior to total RNA extraction using NanoDrop at 414 nm absorbance (ABS_414_). All samples with ABS_414_ > 5 were excluded for further steps.

Total RNA was extracted from 100 μl of plasma using miRNeasy Mini Kit (Qiagen) according to manufacturer’s instructions. Cel-miR-39-3p was added to the plasma before extraction to be used as spike-in extraction control.

### Reverse transcription and cDNA preparation

Reverse transcriptions of RNA extracted from blood samples of the Rwanda recruitment site were performed using the same protocol than used previously for the Belgian cohort^25^. Reverse transcription of RNA was carried out using miRCURY LNA™ Universal RT microRNA PCR (RT-qPCR universal cDNA synthesis Kit II) (Exiqon) following the manufacturer’s instructions, using UniSP6 spike-in as reverse transcription control.

### microRNA profiling

MicroRNA profiling of RNA extracted from blood samples of the Rwanda recruitment site were performed using the same protocol than used previously for the Belgian cohort^25^. Quantitative PCR (qPCR) were performed on custom panels for selected microRNA (Pick-&-Mix microRNA PCR Panel, Exiqon). Controls included inter-plate calibrator UniSP3, UniSP6, negative controls and cel-miR-39. The custom panels were slightly different in our previous study performed on the Belgian-recruited cohort and in the Rwandan-recruited cohort. The custom panel used previously for the Belgian-recruited cohort contains 187 microRNAs (list “Frères et al. 2016” available as Table S1)^25^. The custom panel used on the Rwandan-recruited cohort contains 175 microRNAs (list1 available as Table S1). 165 microRNAs were quantified in common on plasma samples collected on both recruitment sites (list2 available as Table S1). Quantitative PCR were performed on a Taqman 7900HT Real –Time PCR System (Applied Biosystems) according to the manufacturer’s protocol.

### Normalization of microRNA expression

Normalization of microRNA profiling from blood samples of the Rwanda recruitment site were performed using the same method than used previously for the Belgian cohort^25^. Raw Cq values for each sample were normalized to the mean Cq of the 50 most expressed microRNAs of the same dataset. These 50 most expressed microRNAs are different according to the data set analyzed. Results were expressed and analyzed as deltaCq (Cq_sample _− Cq_rmean50MostExpressed_).

### Random forest (RF)

#### Feature selection

The feature selection was performed on 100 random resampling partitions of the design dataset (see next point). The importance of the microRNA was measured using both *Mean Decrease in Accuracy* MDA and *Mean Decrease in Gini* MDG. The combined rank according to MDA and MDG was then used to rank the microRNAs. The stability of the feature selection was measured using the Kuncheva and the Spearman indexes calculated by the package OmicsMarkeR^46^.

#### Building of the random forest models

To identify the best microRNA-based signature, all combinations of the microRNAs selected by the previous step are generated, and their performances in terms of AUC are evaluated using a ten-fold cross-validation on the same design cohort.

The design dataset is randomly divided 50 times into training partition containing 90% of the samples which are used to construct the Random Forest models. The remaining 10% of samples constitutes the testing cohort, and are used for a first test of the models.

For all the signatures, the same partitions were used to construct the RF models using parameters *ntree* = 3000, and $mtry\, = \,\sqrt {number\,of\,miRNAs\,in\,the\,signature}$. The Average AUC on the test partitions is then calculated over all the models and reported as AUC. CohortName.TEST in Table S4.

#### Random forest models validation

The best signatures identified in step 2 (modeling) are next validated on one or several independent dataset(s) that is/are normalized separately from de design dataset used in the steps one and two (feature selection and modeling).

### Datasets

The details of the datasets used for Random Forest procedures in this study are summarized in the Table S3.

## Supplementary information

Supplementary Information.

## Data Availability

The raw and normalized data of the miRNA profiling are available upon request.
